# Supplementary material for: Auxin-inducible degradation of UNC-116 in C. elegans inhibits bidirectional dense core vesicle transport and worm locomotion on different timescales
Source: J Cell Sci. 2026 Mar 25;139(6):jcs264245. doi: 10.1242/jcs.264245 (PMC13070256; doi:10.1242/jcs.264245)
Supplement: Supplementary information [file joces-139-264245-s1.pdf]

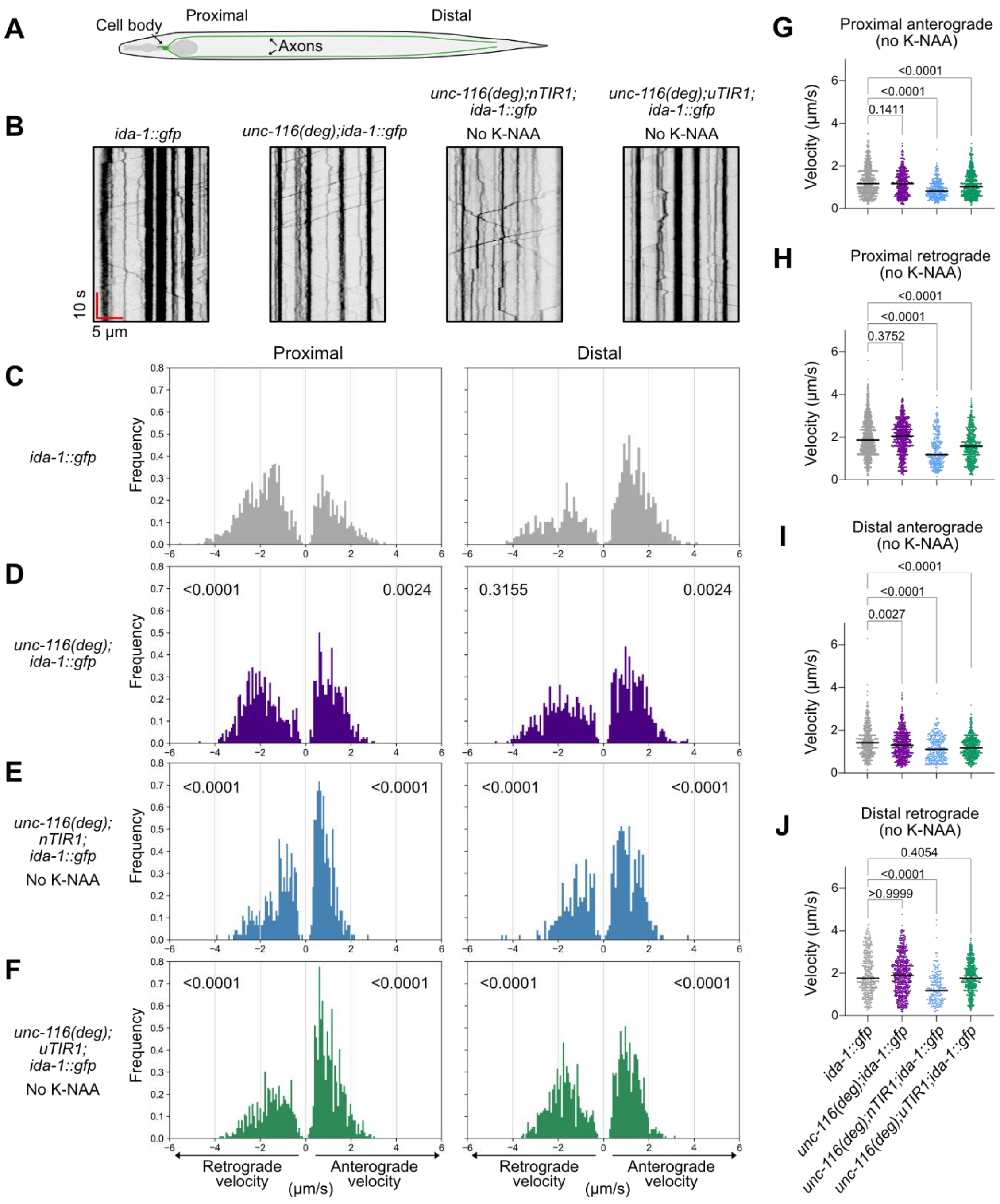

Fig. S1. Effect of UNC-116 degron tagging and background degradation on DCV transport. (A) Schematic of the ALA neuron. (B) Examples of kymographs of DCVs in the proximal ALA neuron with time and distance scales indicated. The head is to the left. (C-F) Distributions of moving DCV track segment velocities in day 1 adults of *ida-1::gfp* (C), *unc-116(deg);ida-1::gfp* (D), *unc-116(deg);nTIR1;ida-1::gfp* (E), and *unc-116(deg);uTIR1;ida-1::gfp* (F) strains grown without K-NAA. The Y-axis displays the probability density function (frequency) with the X-axis showing segment velocity (negative values being retrograde, positive values being anterograde). Kymographs were generated from 16-18 worms: the number of kymographs and segments used to generate velocities are given in table S1. P-values from two-sample K-S tests are shown for movement in both directions, compared to the equivalent data in *ida-1::gfp*. (G-J) The same segment velocities plotted in scatter plots, displaying the median. Strains for all panels are indicated on the X-axis in J, with colour coding the same as in (C-F). P-values from Kruskal-Wallis tests followed by Dunn's post-hoc test are shown, comparing each group to *ida-1::gfp.*

Journal of Cell Science • Supplementary information

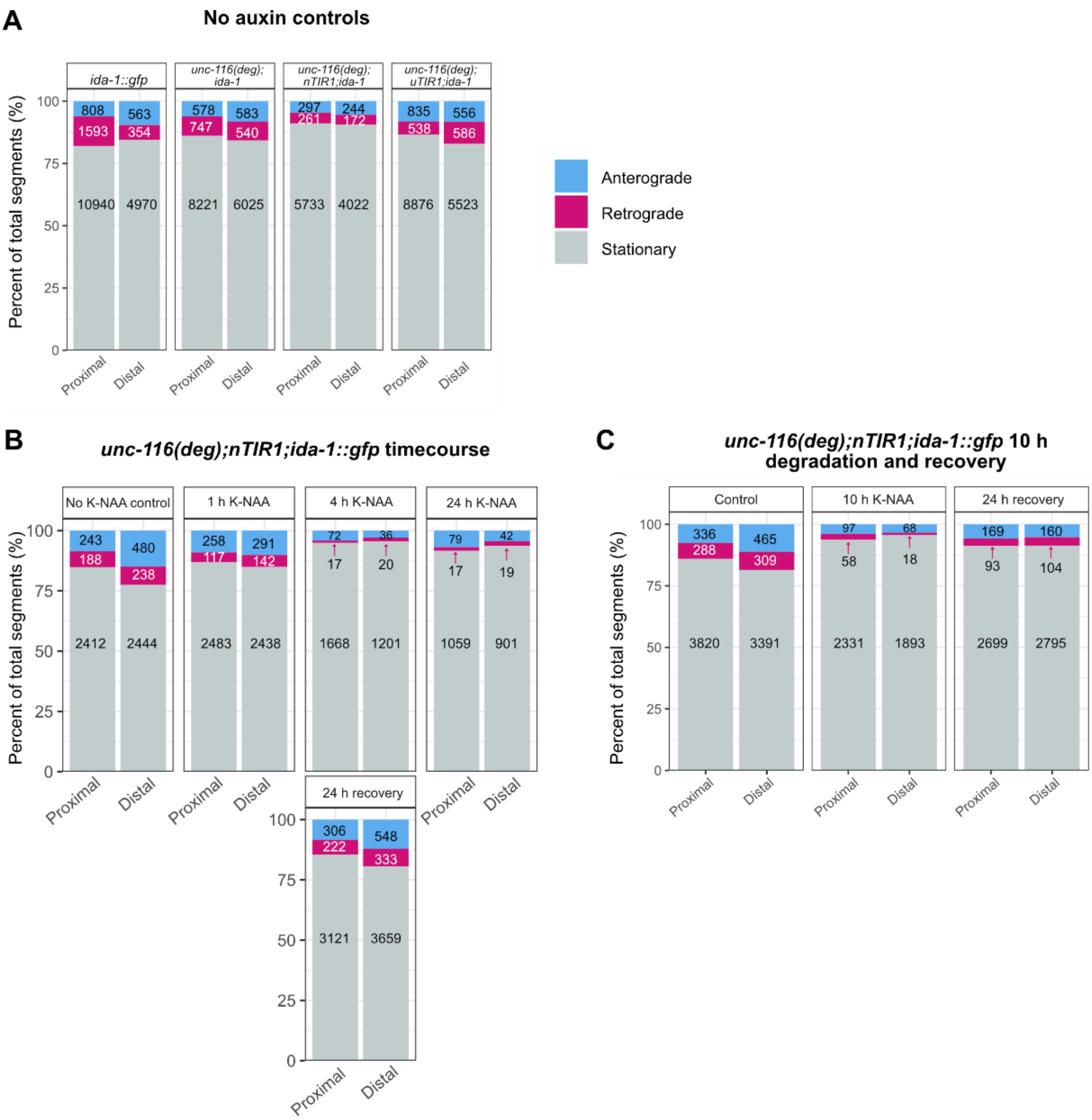

Fig. S2. Segment analysis from DCV tracks in the ALA axon identified from kymographs. Proportion of anterograde, retrograde, and stationary segments identified in untreated strains (data from table S1) (A), *unc-116(deg);nTIR1;ida-1::gfp* treated with K-NAA for indicated times (data from table S2) (B) and *unc-116(deg);nTIR1;ida-1::gfp* treated with K-NAA for 10 h followed by 24 h rescue (data from table S3). Number of segments in each category is indicated on the graphs.

Journal of Cell Science • Supplementary information

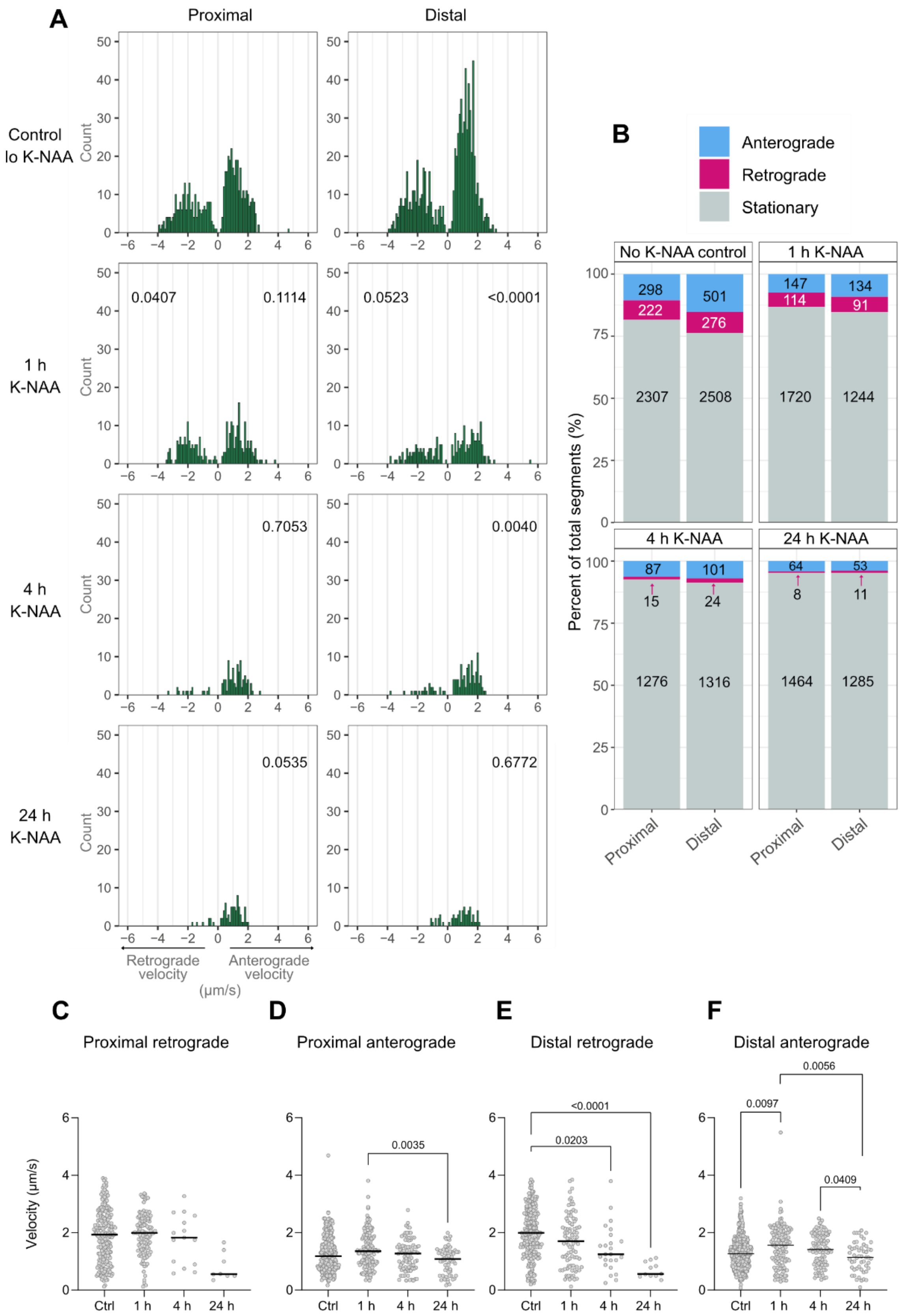

Fig. S3. Both anterograde and retrograde DCV transport rapidly decline after ubiquitous UNC-116 degradation. (A) Velocity distributions of moving DCV track segments after incubation without (control) or plus K-NAA for 1, 4 or 24 hours with the number of moving segments (count) on the Y-axis. Number of worms, kymographs, and segments

Journal of Cell Science • Supplementary information

used to generate velocities are given in table S4. P-values are shown from two-sample K-S tests comparing retrograde and anterograde velocities in each condition and location to the equivalent subset in the untreated worms. The statistical analysis excluded the retrograde data from 4 h and 24 h, due to the limited number of values. (B) Proportion of anterograde, retrograde, and stationary segments identified in *unc-116(deg);uTIR1;ida-1::gfp* treated with K-NAA for indicated times (data from table S4). (C-F) The same data plotted in scatter plots displaying the P-values ≤0.05 from Kruskal-Wallis tests followed by Dunn's post-hoc test are shown.

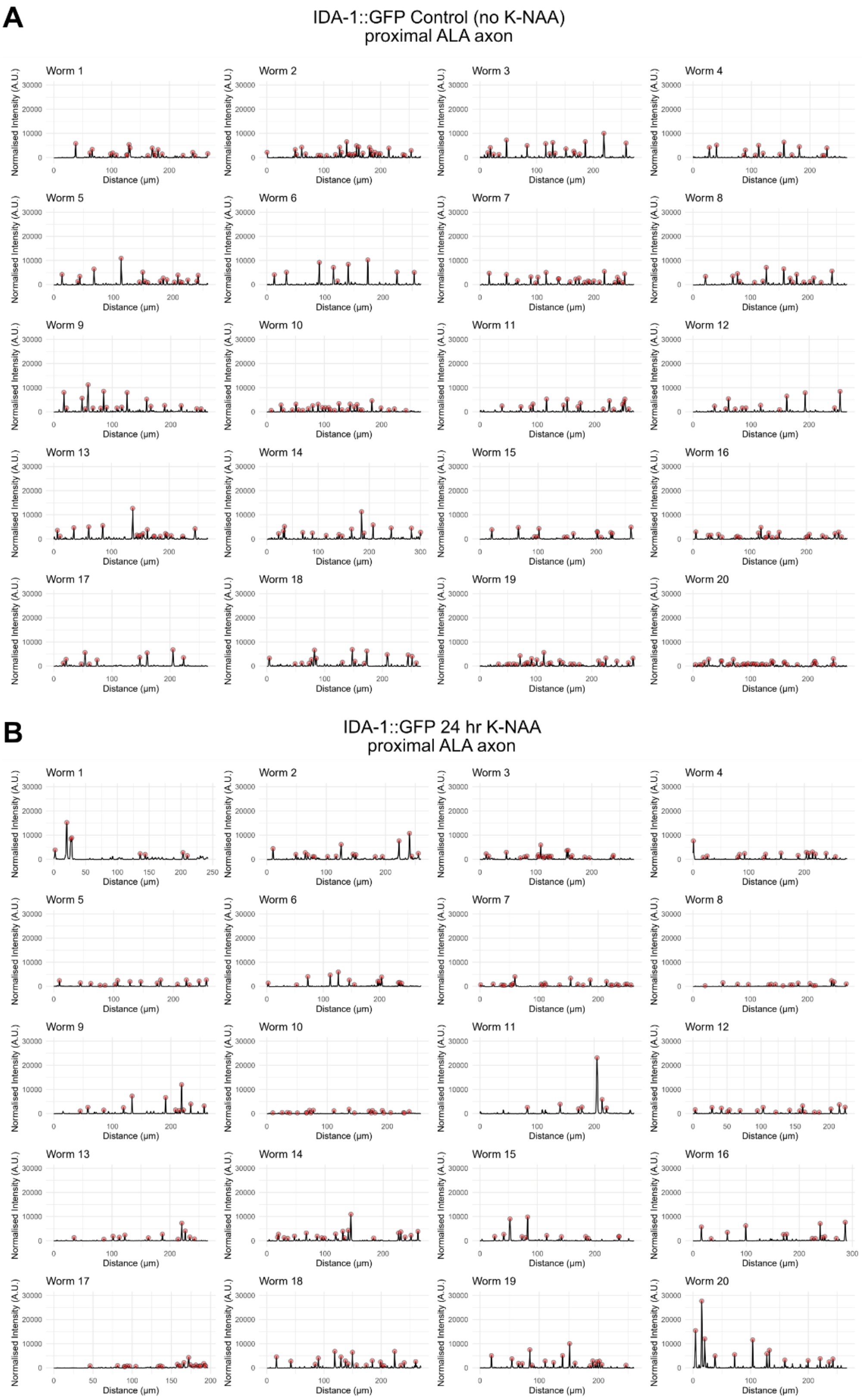

Fig. S4. Intensity profiles of steady-state DCV distribution (IDA-1::GFP) from all proximal ALA axons analysed in fig. 5. Intensity profiles from untreated (control) worms (A), and worms treated with K-NAA for 24 h (B), with the intensity normalised to the median on the Y-axis and distance (µm) on the X-axis.

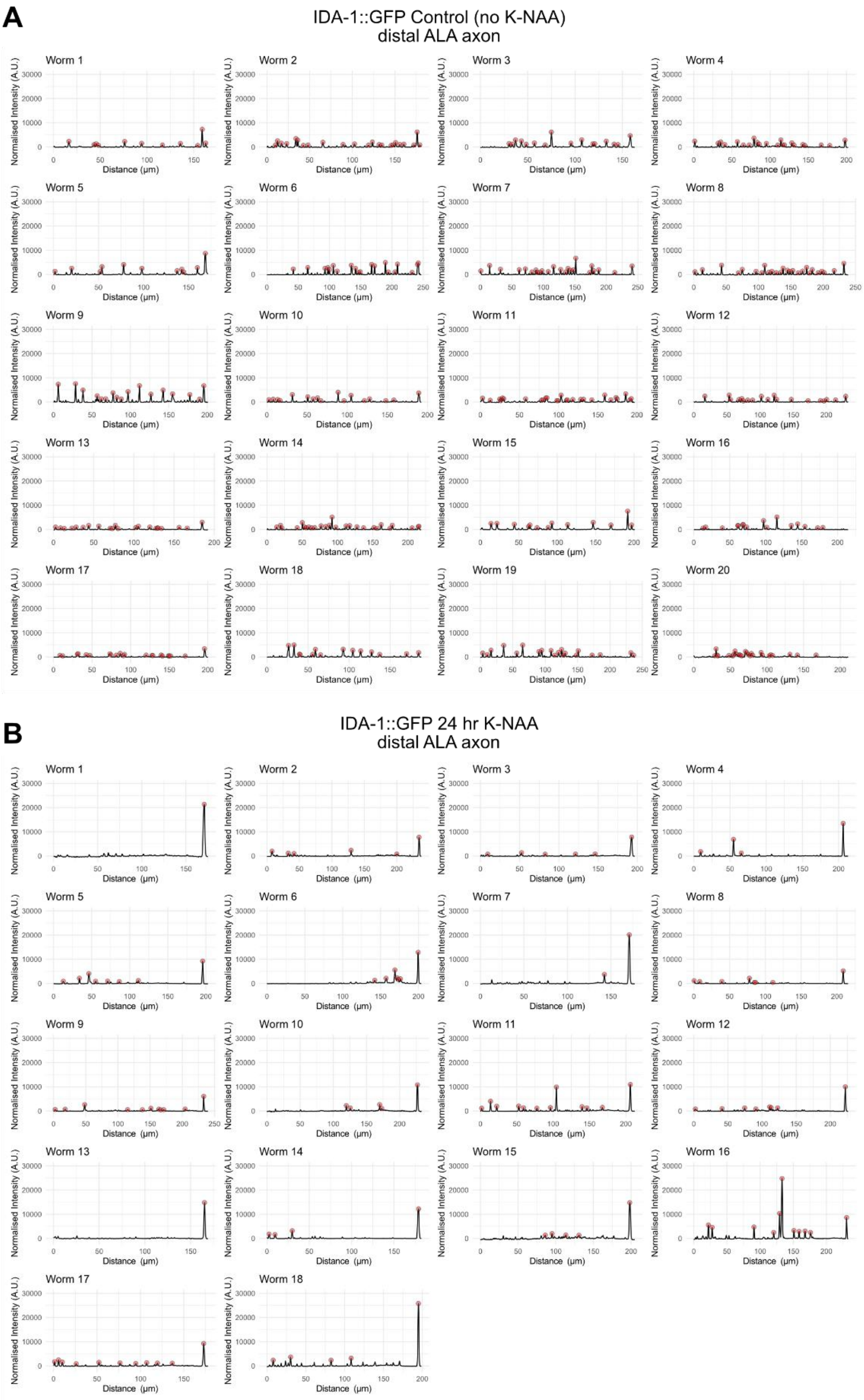

Fig. S5. Intensity profiles of steady-state DCV distribution (IDA-1::GFP) from all distal ALA axons analysed in fig. 5. Intensity profiles from untreated (control) worms (A), and worms treated with K-NAA for 24 h (B), with the intensity normalised to the median on the Y-axis and distance (μm) on the X-axis.

Journal of Cell Science • Supplementary information

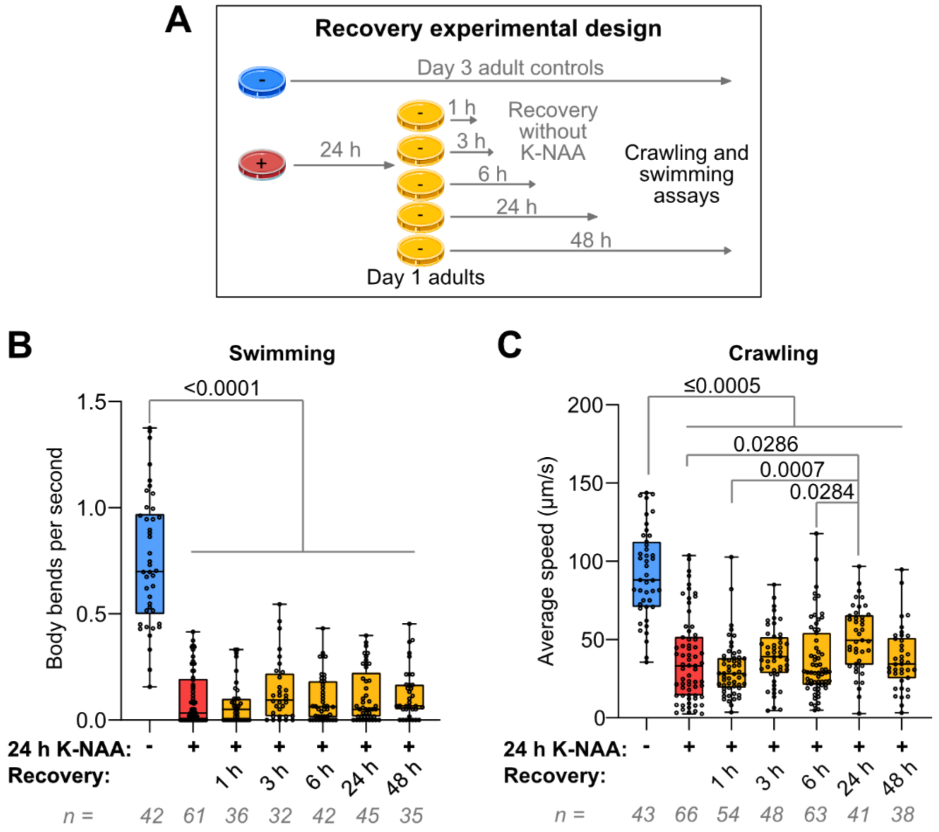

Fig. S6. Poor recovery of locomotion after removal of K-NAA following a 24 hour . Time course analysis of swimming (A) and crawling (B) in *unc-116(deg);uTIR1* adults treated with K-NAA for 24 hours followed by incubation on plates without K-NAA for the time indicated. The number of worms analysed across three independent experiments is given below each graph, with boxes displaying the median and IQR with whiskers showing the data range. Statistical analysis was by Kruskal-Wallis test followed by Dunn's post-hoc test. Only P-values ≤0.05 are indicated on the graphs.

Journal of Cell Science • Supplementary information

5’ -
GGTGATCATTATTCTCTGAAAATGGAGCCGCGGACAATGCCTAAAGATCCAGCCA
AACCTCCGGCCAAGGCACAAGTTGTGGGATGGCCACCGGTGAGATCATACCGG
AAGAACGTGATGGTTTCCTGCCAAAAATCAAGCGGTGGCCCGGAGGCGGCGGC
GTTCGTGAAGTCAGGATCTACATCTGGATCACCATCTAGACTCGAAGAGGAGCT
CCGCAGACGGCTTACCGAACCAGGGGGGACCGGATCCGGGTCCTCGACGTCG
ACGATGGAACCAAGAACTGATGGTGCTGAGTGCGGTGTCCAGGTATTAATTTTC
CCCGCCTAGAT - 3’

Homology arms, AID*, ALFA, Linkers, 27 bp recoded region with silent mutations to prevent re-cutting by sgRNAs

Fig. S7. Donor homology template for CRISPR insertion of AID*-ALFA tag at the *unc-116* locus.

Journal of Cell Science • Supplementary information

Table S1. Summary of DCV motility analysis for strains without K-NAA treatment, from Fig. S1.

Condition	Number of worms	Number of kymographs	Number of tracks	Direction	Number of segments	Mean velocity (µm/s)	Median velocity (µm/s)	Min velocity (µm/s)	Max velocity (µm/s)
ida-1::gfp proximal	17	90	6721	Anterograde	808	1.3	1.2	0.2	3.5
				Retrograde	1593	2.0	1.9	0.2	5.6
				Stationary	10940	-	-	-	-
ida-1::gfp distal	17	69	2967	Anterograde	563	1.5	1.4	0.1	6.3
				Retrograde	354	2.0	1.8	0.3	4.3
				Stationary	4970	-	-	-	-
unc-116(deg); ida-1::gfp proximal	19	70	4806	Anterograde	578	1.2	1.2	0.2	3.1
				Retrograde	747	2.0	2.0	0.2	4.7
				Stationary	8221	-	-	-	-
unc-116(deg); ida-1::gfp distal	18	60	3613	Anterograde	583	1.3	1.3	0.3	3.7
				Retrograde	540	1.9	1.9	0.2	4.8
				Stationary	6025	-	-	-	-
unc-116(deg); nTIR1; ida-1::gfp proximal	17	70	3175	Anterograde	297	0.9	0.8	0.2	2.8
				Retrograde	261	1.4	1.2	0.1	3.9
				Stationary	5733	-	-	-	-
unc-116(deg); nTIR1; ida-1::gfp distal	17	52	2249	Anterograde	244	1.2	1.1	0.1	3.0
				Retrograde	172	1.3	1.2	0.2	4.5
				Stationary	4022	-	-	-	-
unc-116(deg); uTIR1; ida-1::gfp proximal	16	112	5177	Anterograde	835	1.1	1.0	0.1	3.0
				Retrograde	538	1.6	1.6	0.2	3.8
				Stationary	8876	-	-	-	-
unc-116(deg); uTIR1; ida-1::gfp distal	16	79	3390	Anterograde	556	1.2	1.2	0.3	3.2
				Retrograde	586	1.8	1.8	0.2	3.6
				Stationary	5523	-	-	-	-

Journal of Cell Science • Supplementary information

Table S2. Summary of DCV motility analysis in the *unc-116(deg);nTIR1;ida-1::gfp* K-NAA time-course experiment shown in Fig. 4.

Condition	Number of worms	Number of kymographs	Number of tracks	Direction	Number of segments	Mean velocity (µm/s)	Median velocity (µm/s)	Min velocity (µm/s)	Max velocity (µm/s)
Control proximal	12	20	1439	Anterograde	243	1.2	1.2	0.3	2.6
				Retrograde	188	2.2	2.1	0.2	4.2
				Stationary	2412	-	-	-	-
Control distal	10	18	1614	Anterograde	470	1.3	1.2	0.3	3.6
				Retrograde	238	2.0	1.9	0.1	4.6
				Stationary	2444	-		-	-
1 h proximal	13	21	1437	Anterograde	258	1.4	1.4	0.3	3.3
				Retrograde	117	1.8	1.6	0.1	3.8
				Stationary	2483	-	-	-	-
1 h distal	12	21	1461	Anterograde	291	1.5	1.5	0.3	3.1
				Retrograde	142	1.9	1.9	0.3	4.3
				Stationary	2438	-		-	-
4 h proximal	13	19	885	Anterograde	72	1.4	1.4	0.4	2.8
				Retrograde	17	1.5	1.8	0.2	3.6
				Stationary	1668	-	-	-	-
4 h distal	13	19	634	Anterograde	36	1.5	1.6	0.4	2.8
				Retrograde	20	1.5	1.4	0.4	2.8
				Stationary	1201	-		-	-
24 h proximal	13	19	588	Anterograde	79	1.2	1.2	0.2	3.1
				Retrograde	17	1.8	1.7	0.3	3.4
				Stationary	1059	-	-	-	-
24 h distal	12	19	486	Anterograde	42	1.4	1.5	0.1	2.6
				Retrograde	19	1.9	1.8	0.5	4.2
				Stationary	901	-		-	-
Recovery proximal	15	29	1850	Anterograde	306	1.2	1.1	0.2	3.2
				Retrograde	222	1.6	1.5	0.1	3.7
				Stationary	3121	-	-	-	-
Recovery distal	15	27	2310	Anterograde	548	1.4	1.4	0.1	3.0
				Retrograde	333	1.8	1.8	0.2	4.0
				Stationary	3659	-	-	-	-

Journal of Cell Science • Supplementary information

Table S3. Summary of DCV motility analysis in the *unc-116(deg);nTIR1;ida-1::gfp* K-NAA time-course experiment shown in Fig. 8.

Condition	Number of worms	Number of kymographs	Number of tracks	Direction	Number of segments	Mean velocity (μm/s)	Median velocity (μm/s)	Min velocity (μm/s)	Max velocity (μm/s)
Control proximal	12	24	2241	Anterograde	336	1.4	1.4	0.1	2.9
				Retrograde	288	2.1	2.3	0.2	4.3
				Stationary	3820	-	-	-	-
Control distal	12	23	2118	Anterograde	465	1.4	1.3	0.1	6.3
				Retrograde	309	1.8	1.8	0.1	4.8
				Stationary	3391	-	-	-	-
10 h proximal	11	22	1247	Anterograde	97	1.2	1.1	0.4	2.9
				Retrograde	58	1.7	1.6	0.2	3.9
				Stationary	2331	-	-	-	-
10 h distal	11	20	992	Anterograde	68	1.3	1.3	0.2	2.9
				Retrograde	18	1.8	1.5	0.2	4.1
				Stationary	1893	-	-	-	-
Recovery proximal	11	25	1494	Anterograde	169	1.2	1.2	0.3	2.6
				Retrograde	93	1.5	1.4	0.2	4.1
				Stationary	2699	-	-	-	-
Recovery distal	10	21	1549	Anterograde	160	1.1	0.9	0.2	2.6
				Retrograde	104	1.4	1.3	0.3	3.7
				Stationary	2795	-	-	-	-

Journal of Cell Science • Supplementary information

Table S4. Summary of DCV motility analysis in the *unc-116(deg);uTIR1;ida-1::gfp* K-NAA time-course experiment shown in Fig. S3.

Condition	Number of worms	Number of kymographs	Number of tracks	Direction	Number of segments	Mean velocity (µm/s)	Median velocity (µm/s)	Min velocity (µm/s)	Max velocity (µm/s)
Control proximal	12	20	1426	Anterograde	298	1.3	1.2	0.2	4.7
				Retrograde	222	1.9	1.9	0.1	3.9
				Stationary	2307	-	-	-	-
Control distal	12	17	1617	Anterograde	501	1.3	1.3	0.1	3.2
				Retrograde	276	2.0	2.0	0.2	3.9
				Stationary	2508	-	-	-	-
1 h proximal	11	17	996	Anterograde	147	1.4	1.4	0.3	3.8
				Retrograde	114	1.9	2.0	0.1	3.4
				Stationary	1720	-	-	-	-
1 h distal	13	13	741	Anterograde	134	1.5	1.6	0.3	5.5
				Retrograde	91	1.7	1.7	0.1	3.8
				Stationary	1244	-	-	-	-
4 h proximal	13	18	693	Anterograde	87	1.2	1.3	0.3	2.8
				Retrograde	15	1.8	1.8	0.6	3.3
				Stationary	1276	-	-	-	-
4 h distal	13	15	725	Anterograde	101	1.4	1.4	0.4	2.5
				Retrograde	24	1.4	1.2	0.2	3.8
				Stationary	1316	-	-	-	-
24 h proximal	12	19	771	Anterograde	64	1.1	1.1	0.2	2.0
				Retrograde	8	0.8	0.6	0.3	1.7
				Stationary	1464	-	-	-	-
24 h distal	12	18	677	Anterograde	53	1.2	1.1	0.1	2.1
				Retrograde	11	0.7	0.6	0.3	1.1
				Stationary	1285	-	-	-	-

Journal of Cell Science • Supplementary information

Table S5. *C. elegans* strains used in this study

Strain name	Referred to as	Genotype	Source
Bristol N2	*N2*	Wild-type	CGC
BL5752	*ida-1::gfp*	*inIs182 (ida-1p::ida-1::gfp) I; inIs181 (ida-1p::ida-1::gfp) IV*	Zahn et al. (2004)
N/A	*unc-116 mutant*	*unc-116 (rh24sb79) III*	Yang et al. (2005)
OL332	*unc-116(deg)*	*unc-116(uk1[AID::ALFA::unc-116]) III*	This study
JDW225	*uTIR1*	*wrdSi23 [eft-3p::TIR1::F2A::mTagBFP2::AID*::NLS::tbb-2 3'UTR] (I:-5.32)]*	CGC Ashley et al. (2021)
OL334	*nTIR1*	*ukEx31[rab-3p::3xFLAG::TIR1::tbb-2 + (pNU936) unc-119p::unc-119::unc-119u + (pNU3225) NLS::myo-2::GFP]; unc-119(ed3)*	This study
OL370	*unc-116(deg);uTIR1*	*unc-116(uk1[AID::ALFA::unc-116]) III); wrdSi23 [eft-3p::TIR1::F2A::mTagBFP2::AID*::NLS::tbb-2 3'UTR] (I:-5.32).*	This study
OL389	*unc-116(deg);nTIR1*	*unc-116(uk1[AID::ALFA::unc-116] III; ukEx31[rab-3p::3xFLAG::TIR1::tbb-2 + (pNU936) unc-119p::unc-119::unc-119u + (pNU3225) NLS::myo-2::GFP]*	This study
OL390	*unc-116(deg);uTIR1;ida-1::gfp*	*unc-116(uk1[AID::ALFA::unc-116] III); wrdSi23 [eft-3p::TIR1::F2A::mTagBFP2::AID*::NLS::tbb-2 3'UTR] (I:-5.32); inIs182 (ida-1p::ida-1::gfp) I; inIs181 (ida-1p::ida-1::gfp) IV*	This study
OL391	*unc-116(deg);nTIR1;ida-1::gfp*	*unc-116(uk1[AID::ALFA::unc-116] III; ukEx31[rab-3p::TIR1::tbb-2 + (pNU936) unc-119p::unc-119::unc-119u + (pNU3225) NLS::myo-2::GFP]; inIs182 (ida-1p::ida-1::gfp) I; inIs181 (ida-1p::ida-1::gfp) IV*	This study
OL479	*unc-116(deg);ida-1::gfp*	*unc-116(uk1[AID::ALFA::unc-116] III); inIs182 (ida-1p::ida-1::gfp) I; inIs181 (ida-1p::ida-1::gfp) IV*	This study
KP3947	*unc-129p::nlp-21::venus*	*nuIs183 [unc-129p::nlp-21::Venus + myo-2p::GFP].*	CGC
KP3814	*unc-129p::gfp::snb-1*	*nuIs152 [unc-129p::GFP::snb-1 + ttx-3p::mRFP] II.*	CGC
OL501	*unc-116(deg);uTIR1;nlp-21*	*unc-116(uk1[AID+ALFA::unc-116]) III); wrdSi23 [eft-3p::TIR1::F2A::mTagBFP2::AID*::NLS::tbb-2 3'UTR] (I:-5.32); nuIs183 [unc-129p::nlp-21::Venus + myo-2p::GFP].*	This study
OL502	*unc-116(deg);uTIR1;snb-1*	*unc-116(uk1[AID+ALFA::unc-116]) III); wrdSi23 [eft-3p::TIR1::F2A::mTagBFP2::AID*::NLS::tbb-2 3'UTR] (I:-5.32);nuIs152 [unc-129p::GFP::snb-1 + ttx-3p::mRFP] II.*	This study
OL495	*ida-1p::ebp-2::wrmScarlet*	*ukSi012[pQ050; ida-1p::ebp-2::wrmScarlet::unc-54 3'UTR; cb-unc119(+)]V*	This study

Table S6. Antibodies used

Antibody	Host species	Dilution	Source	Product number
TAT1 (Tubulin) mAb	Mouse	1:5000	Keith Gull, University of Oxford	N/A
ALFA 800 CW	Camelid	1:10,000	NanoTag	N1502-Li800-L
Anti-Mouse 680	Donkey	1:10,000	Jackson ImmunoResearch Laboratories	715-625-151

Journal of Cell Science • Supplementary information

Figure 1B

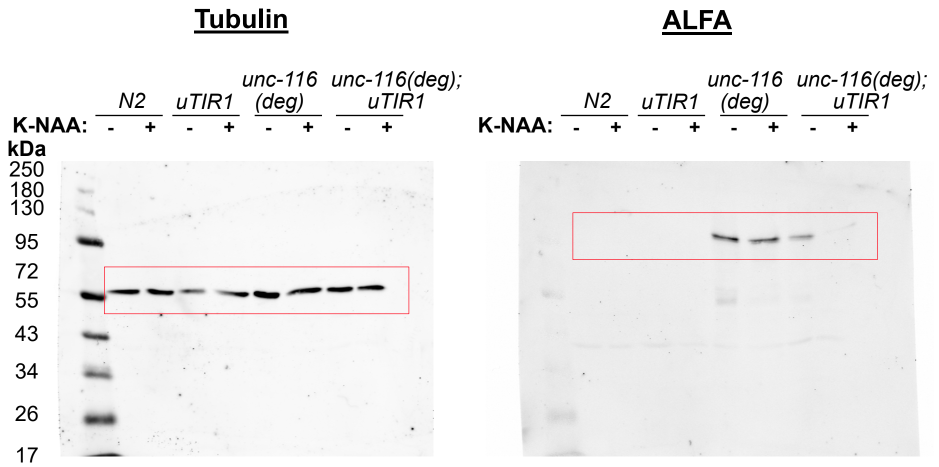

Figure 1C

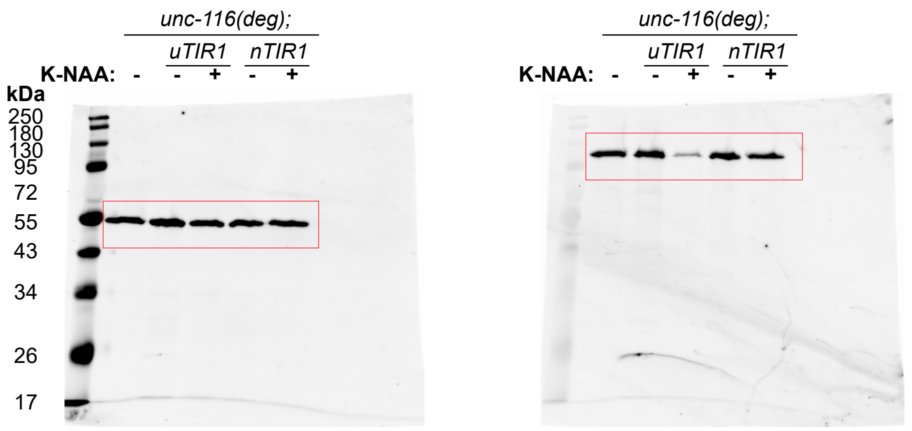

Figure 1D

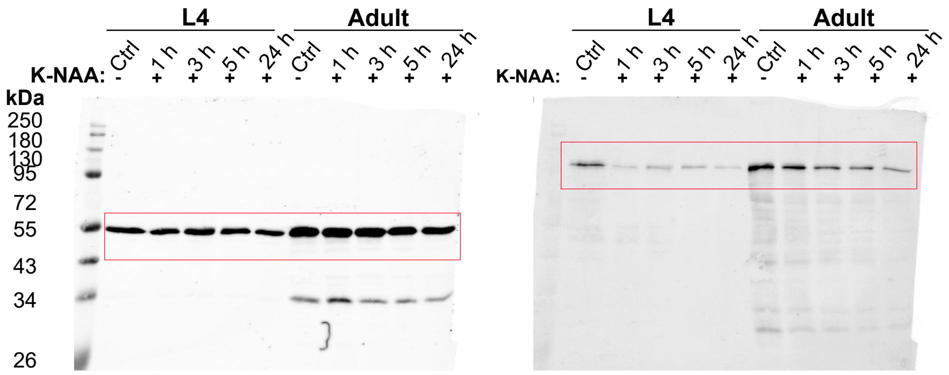

Journal of Cell Science • Supplementary information

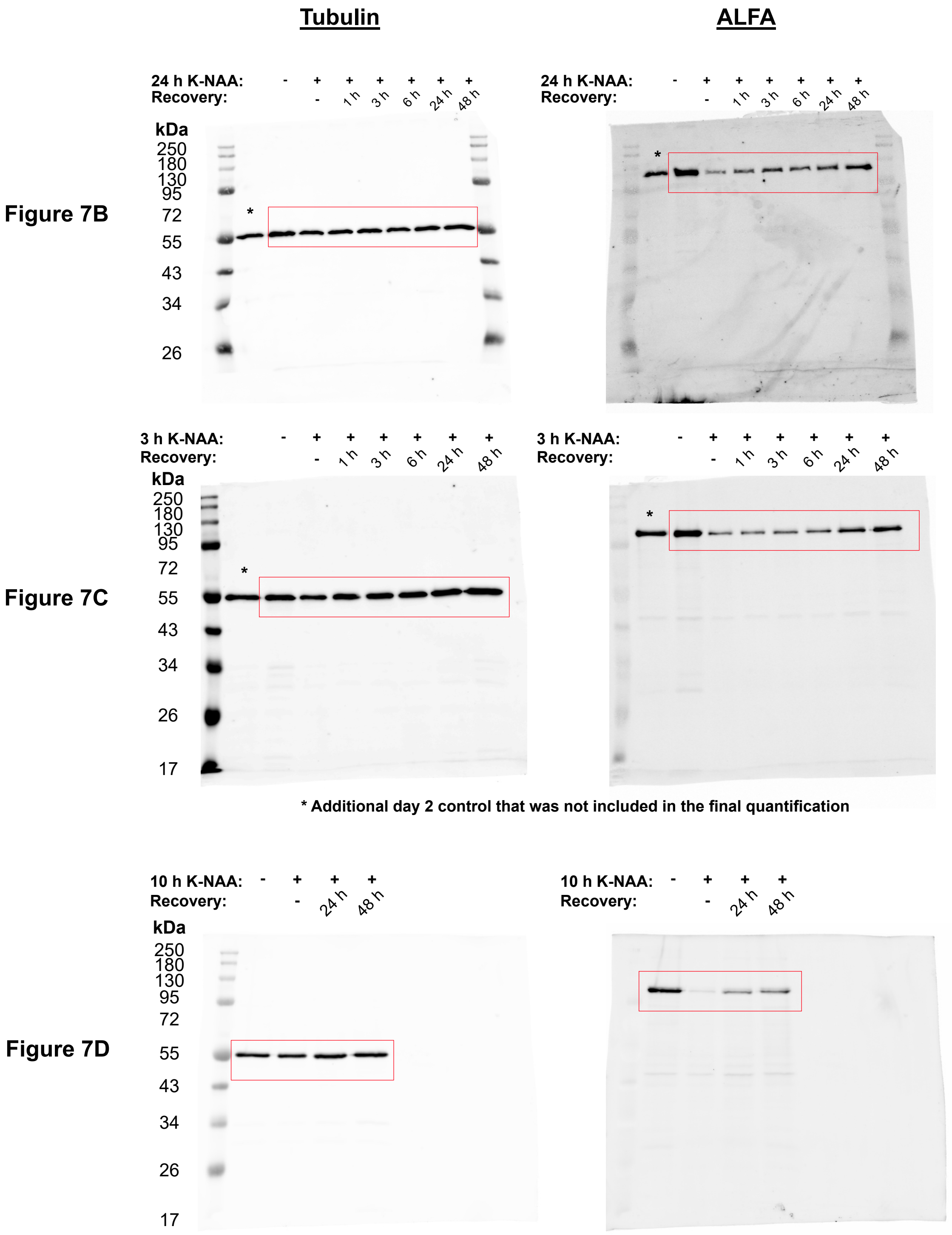

Fig. S8. Blot Transparency.

Journal of Cell Science • Supplementary information

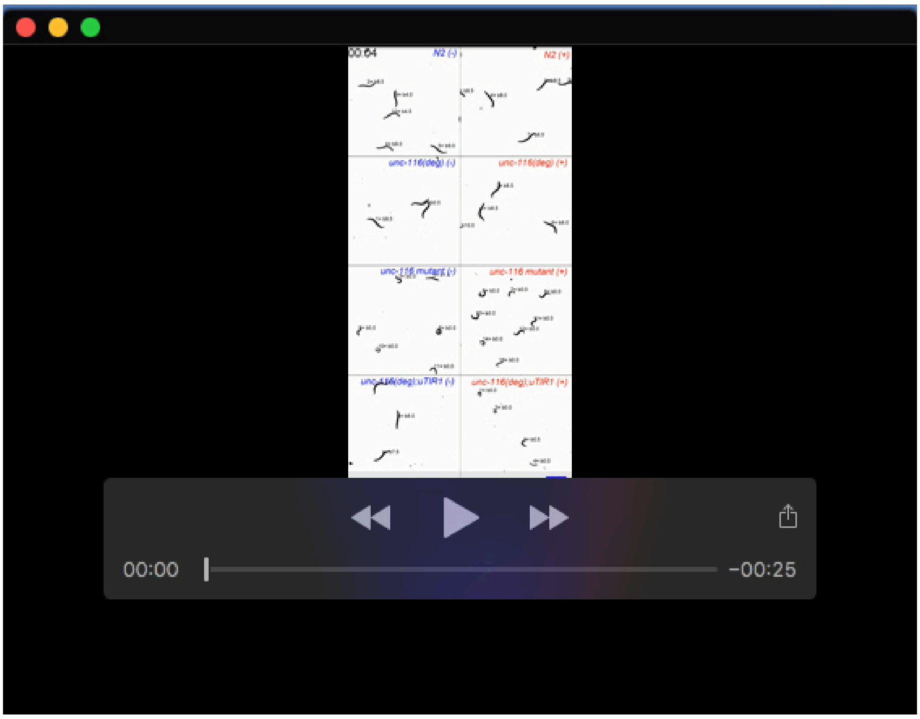

Movie 1. Swimming ability is lost after UNC-116 degradation. Binary masks of indicated worm strains grown ± K-NAA for 72 hours after L1 synchronization are displayed. Swimming/ thrashing ability was analysed by the wrmTrck Fiji plugin, which assigns each worm with a number and counts number of body bends (b). Time (m:s) is indicated. The frame rate is 25 fps. Scale bar = 1 mm.

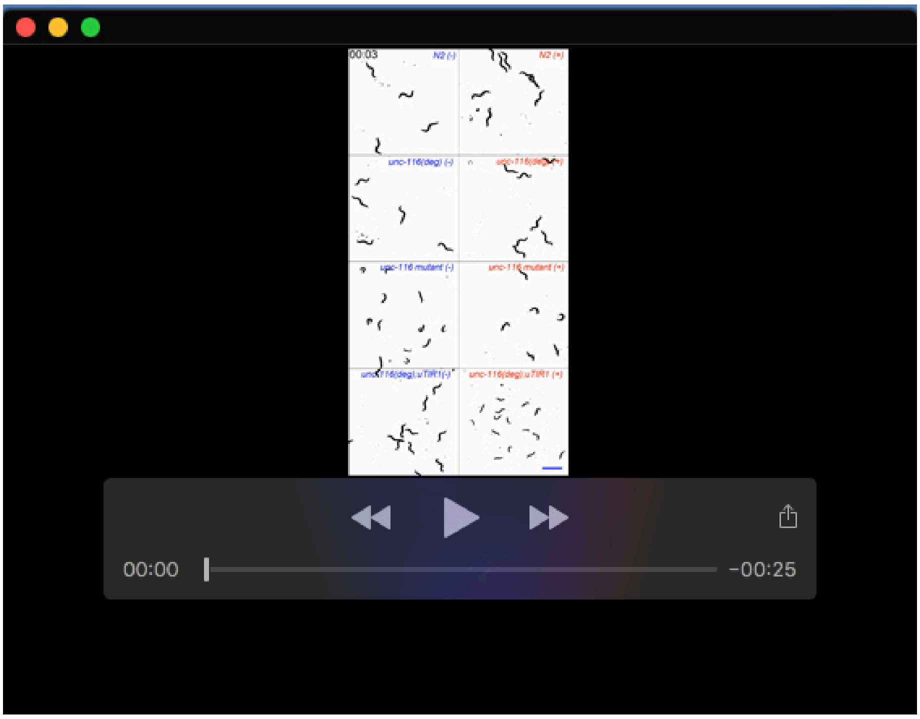

Movie 2. Crawling ability is lost after UNC-116 degradation. Binary masks of indicated worm strains grown ± K-NAA for 72 hours after L1 synchronization are displayed. Time (m:s) is indicated. The frame rate is 25 fps. Scale bar = 1 mm.

Journal of Cell Science • Supplementary information

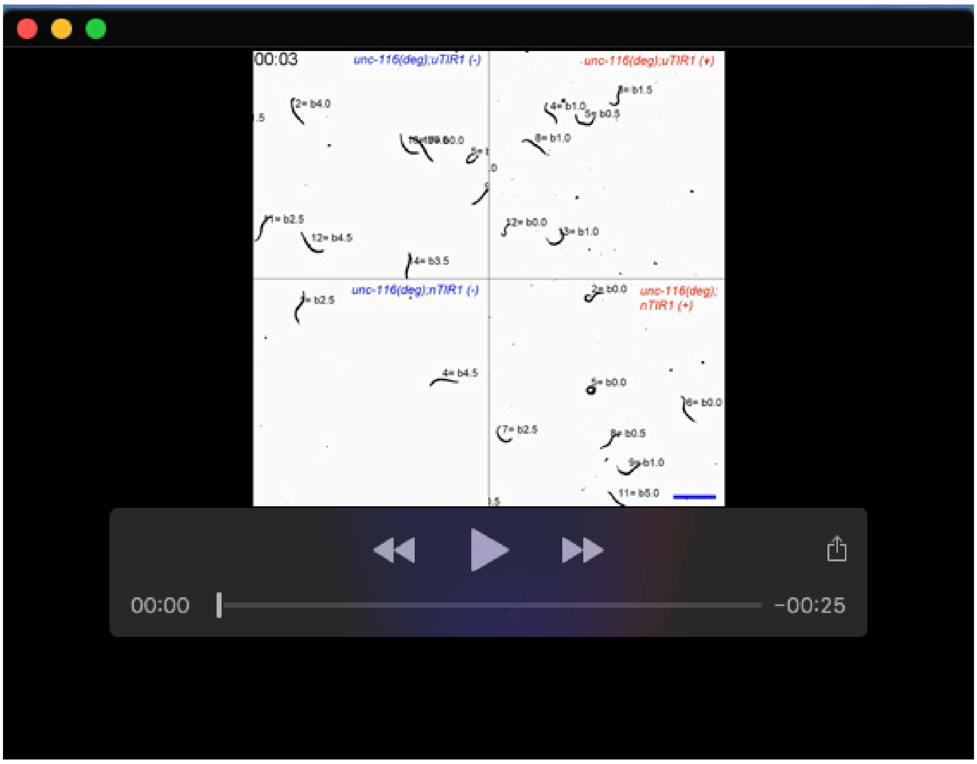

Movie 3. Neuronal and somatic UNC-116 degradation leads to swimming defects. Binary masks of day 1 adults of the indicated strains grown ± K-NAA for 24 hours. Time (m:s) is indicated. The frame rate is 25 fps. Scale bar = 1 mm; b=number of body bends.

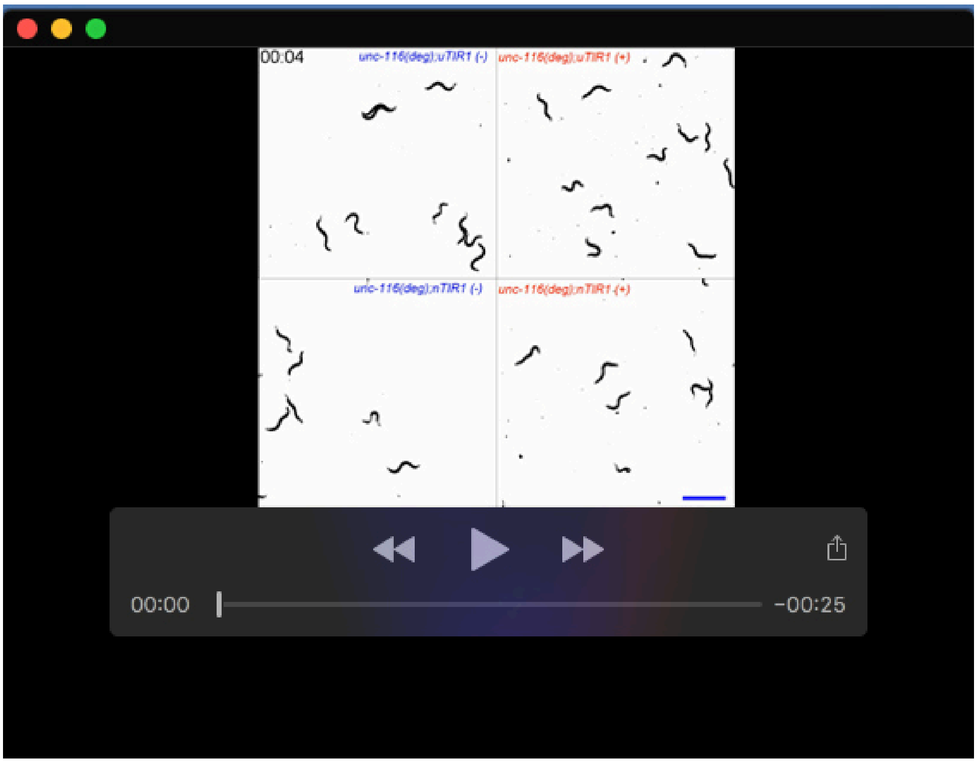

Movie 4. Neuronal and somatic UNC-116 degradation leads to crawling defects. Binary masks of day 1 adults of the indicated strains grown ± K-NAA for 24 hours. Time (m:s) is indicated. The frame rate is 25 fps. Scale bar = 1 mm.

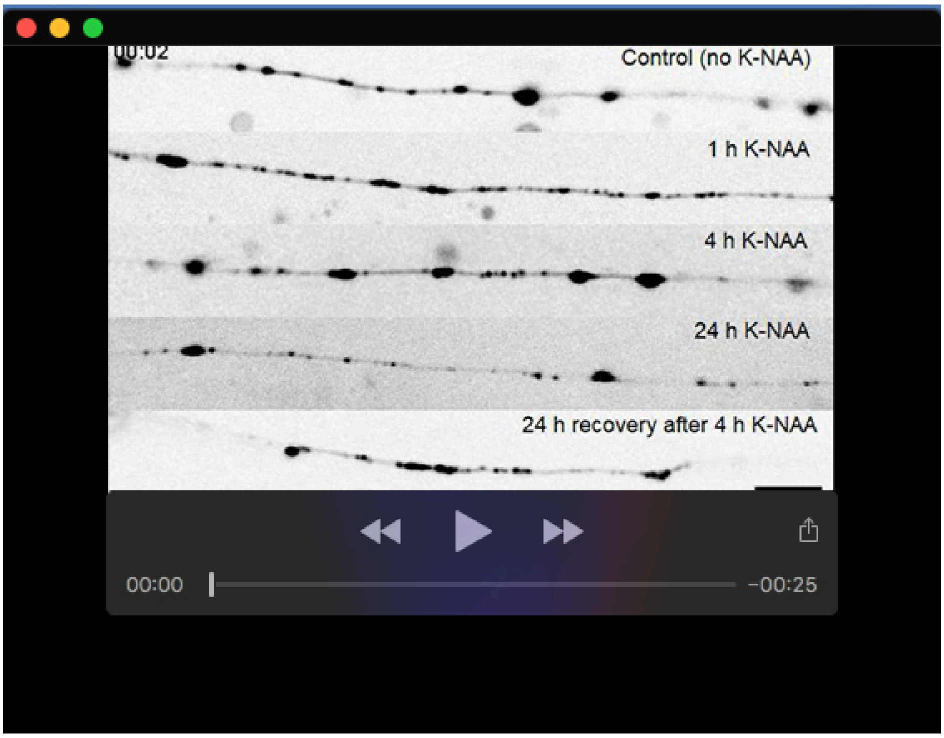

Movie 5. DCV transport in the proximal ALA axon in *unc-116(deg);nTIR1;ida-1::gfp* after periods of K-NAA treatment and recovery. DCV transport is displayed in untreated (control) worms, worms treated with K-NAA for 1, 4 and 24 hours, and worms that have been allowed to recover from K-NAA for 24 hours after 4 hours of treatment. Time (m:s) is indicated, with a frame rate of 10 fps with 184 ms/frame. Scale bar = 5 µm.

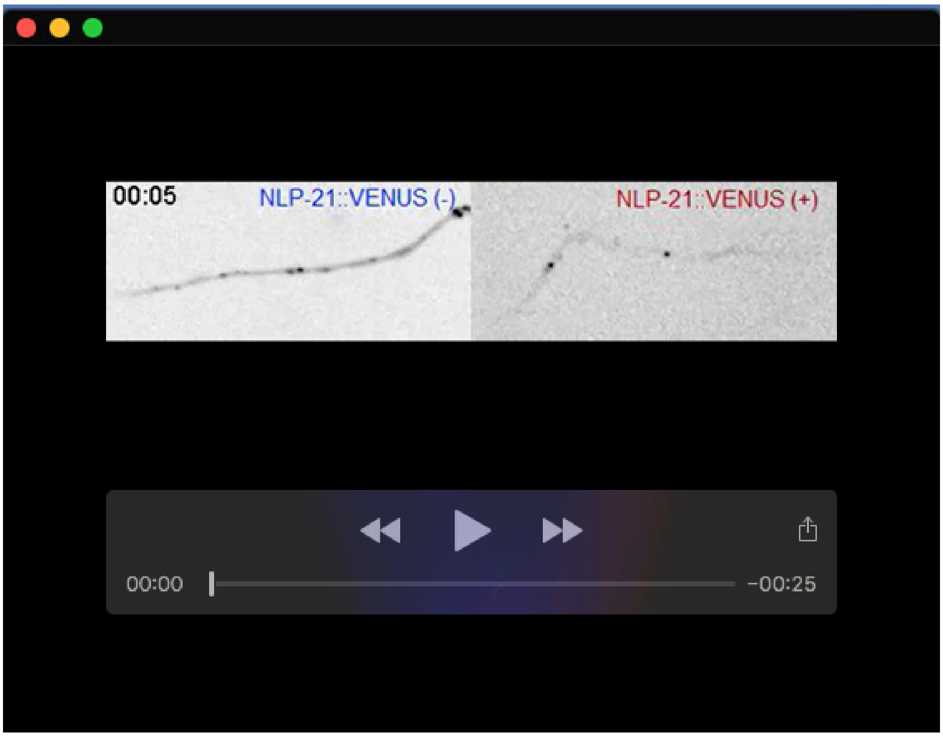

Movie 6. DCV transport in the DB7 axon commisure of untreated *unc-116(deg);uTIR1;nlp-21::venus* worms and worms treated with K-NAA for 24 hours. Movement in untreated (-) worms and K-NAA-treated (+) worms is seen. Time (m:s) is indicated, with a frame rate of 10 fps with 184 ms/frame. Scale bar = 5 µm.

Journal of Cell Science • Supplementary information
